# Architecture and matrix assembly determinants of *Bordetella pertussis* biofilms on primary human airway epithelium

**DOI:** 10.1371/journal.ppat.1011193

**Published:** 2023-02-23

**Authors:** Audra R. Fullen, Jessica L. Gutierrez-Ferman, Rachael E. Rayner, Sun Hee Kim, Phylip Chen, Purnima Dubey, Daniel J. Wozniak, Mark E. Peeples, Estelle Cormet-Boyaka, Rajendar Deora

**Affiliations:** 1 The Department of Microbial Infection and Immunity, The Ohio State University Wexner Medical Center, Columbus, Ohio, United States of America; 2 Department of Veterinary Biosciences, The Ohio State University, Columbus, Ohio, United States of America; 3 Center for Vaccines and Immunity, Abigail Wexner Research Institute at Nationwide Children’s Hospital, Columbus, Ohio, United States of America; 4 Department of Microbiology, The Ohio State University, Columbus, Ohio, United States of America; 5 Department of Pediatrics, The Ohio State University, Columbus, Ohio, United States of America; University of Maryland, UNITED STATES

## Abstract

Traditionally, whooping cough or pertussis caused by the obligate human pathogen *Bordetella pertussis* (*Bp*) is described as an acute disease with severe symptoms. However, many individuals who contract pertussis are either asymptomatic or show very mild symptoms and yet can serve as carriers and sources of bacterial transmission. Biofilms are an important survival mechanism for bacteria in human infections and disease. However, bacterial determinants that drive biofilm formation in humans are ill-defined. In the current study, we show that *Bp* infection of well-differentiated primary human bronchial epithelial cells leads to formation of bacterial aggregates, clusters, and highly structured biofilms which are colocalized with cilia. These findings mimic observations from pathological analyses of tissues from pertussis patients. Distinct arrangements (mono-, bi-, and tri-partite) of the polysaccharide Bps, extracellular DNA, and bacterial cells were visualized, suggesting complex heterogeneity in bacteria-matrix interactions. Analyses of mutant biofilms revealed positive roles in matrix production, cell cluster formation, and biofilm maturity for three critical *Bp* virulence factors: Bps, filamentous hemagglutinin, and adenylate cyclase toxin. Adherence assays identified Bps as a new *Bp* adhesin for primary human airway cells. Taken together, our results demonstrate the multi-factorial nature of the biofilm extracellular matrix and biofilm development process under conditions mimicking the human respiratory tract and highlight the importance of model systems resembling the natural host environment to investigate pathogenesis and potential therapeutic strategies.

## Author summary

Despite widespread vaccination, pertussis or whooping cough caused by the gram-negative obligate human pathogen *Bordetella pertussis* (*Bp*) is resurging in many countries. The mechanisms by which *Bp* infects human hosts, causes disease, and persists in the nasopharynx are ill-defined. We show herein that *Bp* forms aggregates, clusters, and highly structured biofilms colocalized with cilia on well-differentiated and ciliated primary human bronchial epithelial (HBE) cells. The biofilm extracellular matrix (ECM) on HBE cells was heterogenous and consisted of the polysaccharide Bps, extracellular DNA, and bacterial cells arranged in different complexes (mono-, bi-, and tri-partite). Utilizing mutant strains, we discovered positive roles of three *Bp* virulence factors, Bps, filamentous hemagglutinin, and adenylate cyclase toxin in matrix production, cell cluster formation and biofilm maturation. Our study provides a human-relevant model for studying bacterial biofilms, reveals the nature of ECM and bacterial determinants required for biofilm formation on primary human airway cells.

## Introduction

The obligate human pathogen *Bordetella pertussis* (*Bp*) causes whooping cough, also known as pertussis [1–4]. Despite high global vaccine coverage (85–99%), pertussis remains endemic worldwide. Pertussis is notable as a resurging vaccine-preventable disease [5]. Currently, in many countries, acellular pertussis vaccines (aPV) are utilized either as sole vaccines for immunization or as boosters following immunization with whole cell pertussis vaccines. While aPVs effectively prevent severe symptoms of the disease, they do not prevent *Bp* colonization of laboratory animals nor subsequent transmission [6,7].

The incidence of pertussis in young children, adolescents, and adults is severely underestimated [8]. These individuals, in general, exhibit minor (e.g., weight loss and a lingering cough) or no symptoms and yet can serve as carriers and sources of bacterial transmission [9]. A recent study has now unequivocally shown that recent pertussis resurgence in many countries is the result of asymptomatic infections [10]. Moreover, asymptomatic colonization of humans by *Bp* has been directly demonstrated [9].

We propose that *Bp* biofilms play critical roles in its survival and establishment of persistent infection in the human respiratory tract [11–14]. Biofilms are defined as aggregative communities of sessile bacteria encased in an extracellular matrix (ECM) and represent approximately 80% of all chronic microbial human infections. Depending on the microbial species, biofilm ECM on artificial surfaces classically consist of exopolysaccharides, extracellular DNA (eDNA), proteins, lipids, and other polymers [15]. We and others have shown that *Bp* forms biofilms on multiple abiotic surfaces [14,16,17]. These *Bp* biofilms are covered by an opaque ECM composed of eDNA and the polysaccharide Bps, belonging to the poly-β-1,6-N-acetyl-D-glucosamine (PNAG/PGA) family of polysaccharides [14,18]. Mutant *Bp* strains that do not produce Bps, or the protein filamentous hemagglutinin (FHA) are deficient in biofilm formation on abiotic surfaces [18,19]. Another *Bp* protein, adenylate cyclase toxin (ACT) was shown to inhibit *Bp* biofilm formation on these surfaces [16].

*In vitro* biofilm models utilizing abiotic surfaces are heavily dependent on growth media and laboratory conditions and may not reveal all aspects of bacteria-host interactions. We developed respiratory tract mouse models for studying *Bp* biofilm formation. *Bp* biofilms adherent to ciliated respiratory epithelium were found to be (i) encased in Bps, (ii) dissolved by treatment with DNase I, and (iii) dependent upon the production of Bps and FHA [18–20]. The contribution of ACT in respiratory tract biofilm formation is yet to be determined.

*Bp* does not have an environmental or animal reservoir [21]. While mice serve as accessible, cost-effective, and powerful surrogate models for biofilm research, they do not reproduce the full range of disease. There are several differences in the respiratory tract organs between mice and humans, which makes inferences from animal studies difficult to interpret in the context of human infections. Specifically, variations exist in mouse vs. human architectural organization of lungs including the number of bronchioles, branching patterns, and cellular composition [22,23]. Therefore, to fully understand the biofilm development and pathogenesis of obligate human pathogens, there is a critical need for model systems that replicate the human respiratory tract environment.

Towards this goal, we developed an *ex vivo* biofilm formation model by utilizing primary human bronchial epithelial (HBE) cells grown at the air-liquid interface. We report that on HBE cells, *Bp* forms structured biofilms which are encased in an ECM composed of Bps and eDNA. Using specific staining reagents, we detected several morphologically distinct complexes within biofilms between *Bp* cells and ECM components. Finally, using isogenic mutant strains, we report that Bps and FHA are critical for *Bp* adherence to and biofilm formation on HBE cells. Surprisingly, we found that ACT was required for mature biofilm formation on HBE cells, a phenotype different from previous reports using abiotic surfaces [16].

## Results

### *Bp* infects HBE cells

Primary bronchial epithelial cells isolated from human donor lung biopsies were cultured, expanded, and differentiated at air-liquid interface on Transwell filters, as described in Materials and Methods. Briefly, these cells had a polarized, pseudostratified, multi-layered epithelium, consisted of ciliated (S1A–S1C and S1E Fig) and mucus-producing goblet cells lining the luminal surface (S1D and S1F Fig) and intermediate and basal cells (S1 Fig). Additionally, these cells produce the tight junction protein zonula occludens-1 (ZO-1) (S1E Fig) and possess high trans-epithelial electrical resistance (TEER) indicative of high epithelial cell barrier integrity and permeability (Table 1).

**Table 1:** HBE culture characteristics upon *Bp* infection. CBF was quantified from slow-motion capture of 240 frames-per-second. TEER was measured using an epithelial voltohmeter, with 300 Ohms·cm^2^ as the cut-off for acceptable epithelium integrity. Percent LDH was quantified from basal supernatants. Percent acetylated-α-tubulin was quantified from Fig 4B. Mean values are represented, with standard error of the mean values in parentheses. Data represent at least two independent experiments. Significance was calculated by using one-way ANOVA. *, *p* < 0.05 compared to uninfected HBE cells; ^#^, *p* < 0.05 compared to HBE cells infected with WT.

Strain	CBF	TEER	% LDH	% α-tubulin
uninfected	5.85 (0.38)	405 (58)	4.43 (0.19)	25.091 (1.28)
WT	9.35 (0.85)*	200 (17)*	10.18 (0.2)*	31.92 (7.81)
Δ*bpsA-D*	8.18 (0.77)	351 (31)^#^	7.9 (0.58)*^,#^	16.35 (0.81)
Δ*fhaB*	11.4 (1.13)*	264 (7)*	8.2 (0.49)*^,#^	24.47 (4.56)

**Fig 1:**
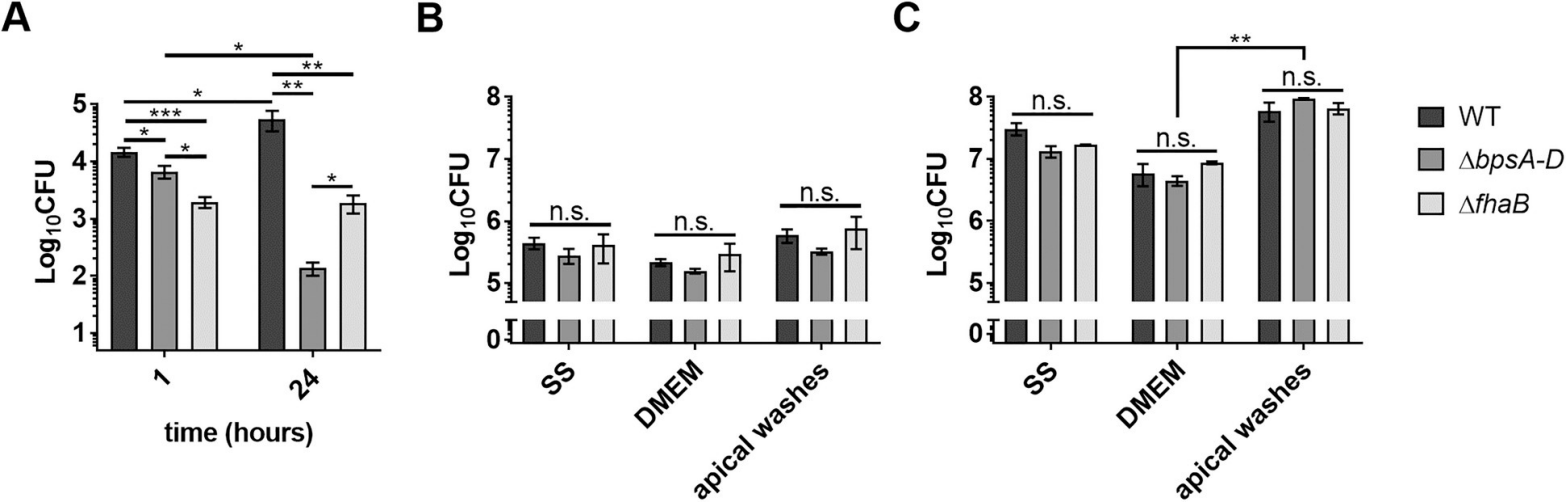
Bps and FHA promote infection of HBE cells. (A) CFUs of WT, Δ*bpsA-D*, and Δ*fhaB* strains after 1 and 24 h of infection. Mean values of four biological replicates in technical duplicate are presented with standard error of the mean. Statistical differences were assessed by two-way ANOVA. *, *p* < 0.05; **, *p* < 0.005; ***, *p* < 0.0005. (B and C) Bacterial strains were added to empty wells (without Transwells) of a 24-well plate in either SS, DMEM, or apical HBE washes. After 1 h (B) or 24 h (C), CFUs were enumerated on BG agar. Mean and standard error of the mean of three biological replicates in technical duplicate are presented. Statistical differences were assessed by two-way ANOVA. **, *p* < 0.005; n.s., not significant.

We apically infected HBE cultures with the WT *Bp* strain (Bp536) at a MOI of 10. One hour later, non-adherent bacteria were washed off and remaining bacteria were enumerated either immediately or after 23 h. As expected, WT bacteria attached to HBE cells after 1 h, and grew 3.7-fold after 24 h (Fig 1A). Previously, we showed that Bps is required for early colonization of mouse lungs [18,24]. Additionally, FHA promotes *Bp* attachment to lung epithelial cells [25,26]. Therefore, to determine the roles of Bps and FHA in *Bp* infection of HBE cells, we infected these cultures with isogenic mutant strains containing in-frame deletions in the *bpsA-D* locus (Δ*bpsA-D*), which encodes for the Bps biosynthesis machinery, and the *fhaB* gene, which encodes for the precursor of FHA (Δ*fhaB*). After 1 h, compared to the WT strain, there was an approximate 2.2-fold and 7.5-fold decrease in colony forming units (CFUs) recovered from Δ*bpsA-D* and Δ*fhaB* mutants, respectively (Fig 1A). These results suggest that both Bps and FHA promote early *Bp* attachment to HBE cells. After 24 h, compared to the WT strain, there was an approximate 400-fold and 30-fold decrease in the CFUs recovered of the Δ*bpsA-D* and Δ*fhaB* mutants, respectively (Fig 1A). To rule out the possibility that the observed phenotypic defects of the mutant strains was due to reduced growth in the media used for infecting HBE cultures, we grew each strain for 1 and 24 h in Stainer-Scholte (SS; *in vitro* growth medium for *Bp*) [27], Dulbecco’s Modified Eagle Medium (DMEM)(used for apical HBE infection), or apical washes obtained from uninfected HBE cultures. No significant differences in the growth among the three strains were observed in any of the culture medium (Fig 1B and 1C). Interestingly, compared to growth in DMEM, a significant increase (~10-fold) in bacterial growth in apical washes was observed for all strains after 24 h, suggesting that apical washes collected from HBE cultures may provide nutrients for better *Bp* growth (Fig 1C). Taken together, these results demonstrate that (a) *Bp* efficiently attaches to and infects HBE cells; (b) the presence of FHA and Bps contributes to efficient infection of HBE cells; and (c) airway surface liquid provides an environment that supports *Bp* growth.

### Impact of *Bp* infection on bronchial epithelium integrity, cilia beating, and cytotoxicity

**Fig 2:**
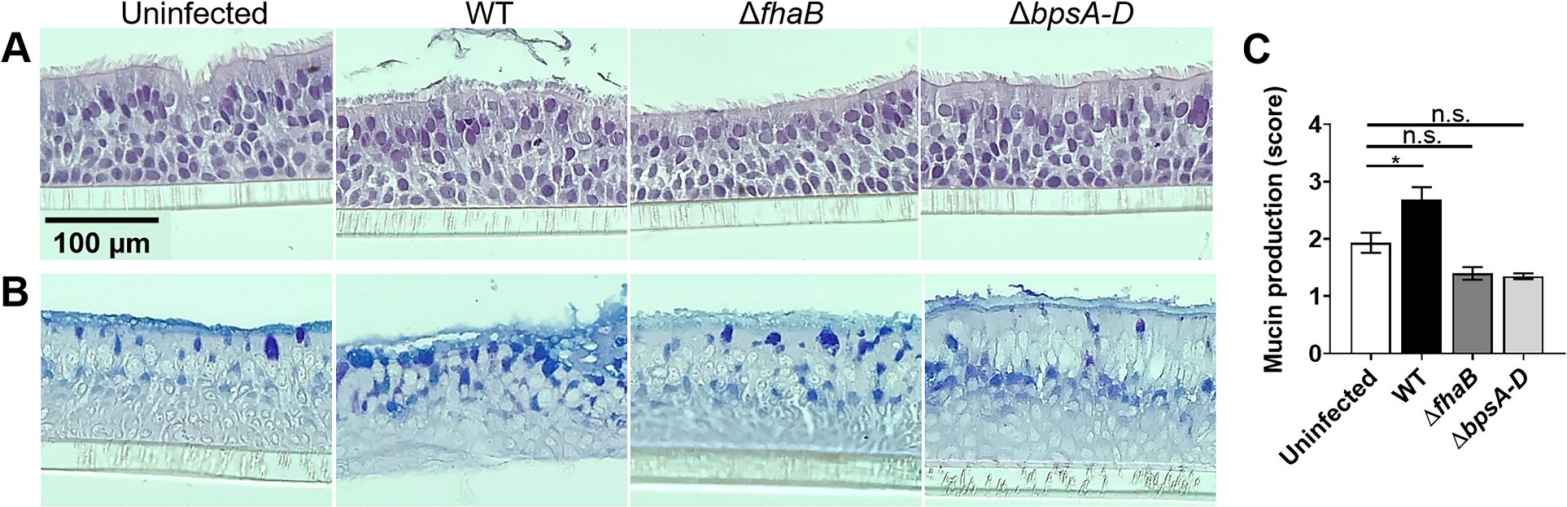
*Bp* induces mucin secretion in HBE cells. HBE cells were infected as described in the ‘Materials and Methods’ at an MOI of 10, for 24 h with different *Bp* strains. Fixed cells were, sectioned and stained with (A) Hematoxylin & Eosin, or (B) PAS/AB. (C) Semi-quantification of mucin production from PAS/AB-stained images. Images are representative of two biological replicates. Statistical differences were assessed by One-way ANOVA. *, *p*≤0.05; n.s., not significant.

Compared to uninfected HBE cells, there were no observable morphological changes (changes in epithelium thickness, and ciliated cells) because of infection with either the WT or the mutant strains (Fig 2A). Mucin secretion, however, was significantly increased upon infection with the WT strain, but not with *ΔfhaB* or *ΔbpsA-D* strains (Fig 2B and 2C). Compared to uninfected HBE cells, the ciliary beat frequency (CBF) of HBE cells infected with WT bacteria and the Δ*fhaB* mutant was significantly increased (Table 1). However, infection with the Δ*bpsA-D* mutant did not significantly alter CBF.

Infection with WT bacteria or the Δ*fhaB* mutant resulted in a significant drop in TEER (Table 1), suggesting a loss of tight junction integrity and barrier function, leading to compromised epithelium and therefore reduced defense against *Bp*. Conversely, the TEER of HBEs infected with the Δ*bpsA-D* strain was not significantly different compared to uninfected HBE cultures (Table 1). These results suggest loss of tight junction integrity upon infection with the Δ*fhaB* mutant but not with the Δ*bpsA-D* strain. *Burkholderia cenocepacia* (K56-2 strain), a Gram-negative bacterium (MOI = 1), was used as a positive control and significantly reduced TEER (<100 Ohms.cm^2^; *p*<0.00004) after 48 h.

Release of lactate dehydrogenase (LDH) is an indicator of cellular cytotoxicity [28]. Compared to uninfected HBE cells, infection with all three *Bp* strains significantly increased LDH released in the basal media. However, compared to infection with the WT strain, infection with either the Δ*bpsA-D* or Δ*fhaB* mutant resulted in the release of significantly lower levels of LDH (Table 1). Together, these data show that *Bp* induces mucin secretion, alters cilia beating, and triggers cytotoxicity in HBE cultures and that the presence of FHA and Bps on the cell surface differentially impacts these properties. However, the observed changes in the HBE cells following infection with the mutant strains could also be due to the reduced numbers of mutant bacteria associating with the HBE cells.

### *Bp* forms biofilms on HBE cells

**Fig 3:**
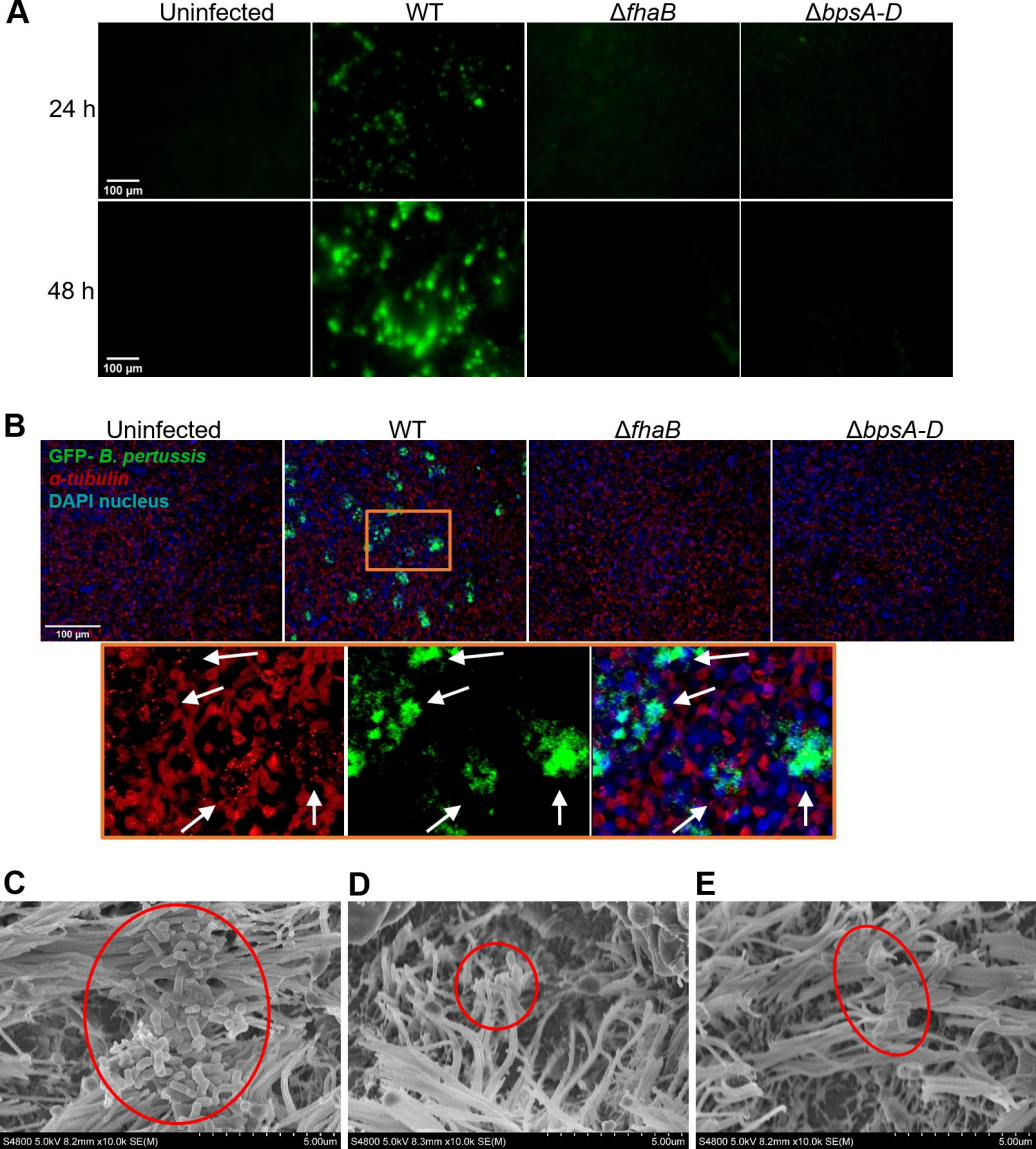
*Bp* forms aggregates and clusters on HBE cells. (A) Representative live cell images of GFP-labeled bacteria on apical surface of HBE cells after 24 and 48 h (10X objective). (B) Immunofluorescent staining of α-tubulin (cilia) and DAPI (nuclei) on fixed HBE cells infected with GFP-labeled bacteria after 48 h (10X objective). Zoomed in image of HBE cells infected with the WT strain showing colocalization of bacterial aggregates with punctate acetylated-α-tubulin staining (arrow). (C-E) Scanning electron microscopy of *Bp* strains 24 h after infection of HBE cells. All images are representative of three biological replicates.

To establish a *Bp* biofilm model, HBE cultures were infected with green fluorescent protein (GFP)-expressing WT, Δ*fhaB*, and Δ*bpsA-D* strains. Aggregates of the WT strain were observed by live-cell imaging at 24 h post-infection, which increased to large biofilm-like clusters at 48 h post-infection (Fig 3A). Since attachment of *Bp* to ciliated respiratory tract mucosa is critical for its pathogenesis [26,29,30], we next determined if the observed bacterial aggregates colocalized with ciliated cells. Infected HBE cells were fixed and stained for acetylated-α-tubulin. WT bacteria colocalized with ciliated cells as shown by the punctate α-tubulin staining in the regions where bacterial aggregates were observed (Fig 3B and S2). In comparison, live cell-imaging of HBE cells infected with either the Δ*bpsA-D* or Δ*fhaB* mutant strain showed very little to no green fluorescence either at 24 or 48 h post-infection (Fig 3A and 3B), indicating the absence of bacterial aggregates/biofilms. Infection with either of the mutant strains did not reveal any significant differences in the relative levels of cilia staining with α-tubulin (Table 1).

To independently confirm the presence of aggregates and biofilm clusters on cilia, infected HBE cells were visualized by scanning electron microscopy (SEM). WT bacteria were colocalized with cilia and were present as large clusters of bacterial cells which are typical of morphological structures observed for bacterial biofilms (Fig 3C). However, following infection with either the Δ*bpsA-D* or Δ*fhaB* strains, few areas on HBE cells harbored bacteria. Only very small aggregates comprised of few bacterial cells were detected in one or two regions (Fig 3D and 3E). In combination, these results demonstrate that *Bp* biofilms on HBE cells colocalize with cilia. Additionally, our data suggest that the absence of FHA and Bps results in a severe defect in the ability of *Bp* to form biofilms/aggregates on airway epithelia.

### Structure and ECM composition of *Bp* biofilms formed on HBE cells

**Fig 4:**
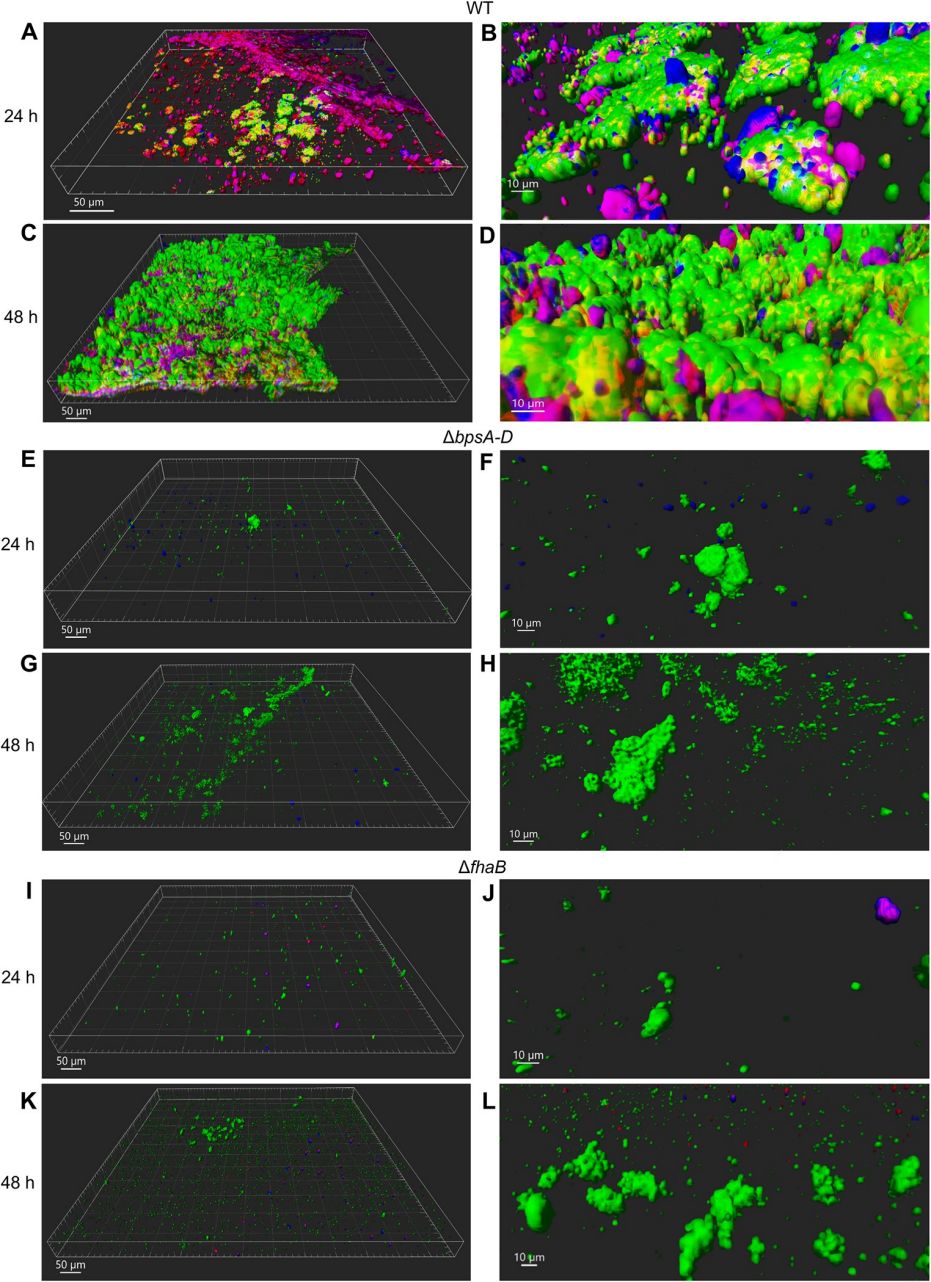
Visualization of *Bp* biofilms and ECM components on HBE cells. HBE cells infected with GFP-expressing WT (A-D), Δ*bpsA-D* (E-H), or Δ*fhaB* (I-L) strains for 24 h (A-B, E-F, I-J) or 48 h (C-D, G-H, K-L) were fixed and stained for Bps (α-PNAG, red) and eDNA (α-dsDNA, blue), then imaged using an Olympus FV3000 confocal microscope (20X objective). B, D, F, H, J, and L are zoomed in images of A, C, E, G, I, and K, respectively. Z-stacks were acquired at 1-μm intervals. IMARIS software was used to produce a shading picture of the biofilms.

Structures of bacterial biofilms and the composition of ECM covering biofilms can vary greatly depending on the type of organism, growth surfaces, and the environment [31]. While considerable information is available from studies of abiotic biofilms, the ECM of bacterial biofilms formed on primary well-differentiated human cells remains poorly characterized. Therefore, HBE cultures were infected with GFP-expressing *Bp* (WT, Δ*bpsA-D*, Δ*fhaB*), stained with specific antibodies to detect Bps (red) and eDNA (blue), and visualized by confocal laser scanning microscopy (CLSM). Three-dimensional reconstructions of z-section image stacks created by IMARIS software revealed large irregularly shaped aggregates of WT bacteria at both 24 and 48 h post-infection (Fig 4A–4D). After 24 h, bacterial cells were found to be present in focal clusters (Fig 4A and 4B), with large areas of the HBE surface devoid of bacterial cells. However, after 48 h, biofilms appeared as mats of tightly packed bacterial cells which occupied many areas of the HBE surface (Fig 4C and 4D).

Analyses of WT biofilm architecture by COMSTAT 2.0 [32] showed a considerable increase in the biofilm biomass from 24 h to 48 h post-infection (Table 2). Moreover, biofilms of WT bacteria became more compact and homogeneous over time with the roughness coefficient decreasing from 24 h to 48 h (Table 2). The average particle size increased from 24 h to 48 h (Table 2). In combination, these results demonstrate that WT *Bp* forms structured biofilms on HBE cells.

**Table 2:** COMSTAT analysis of CLSM images. Values correspond to Fig 4A–4L, 5C, and 5D. Mean values are represented, with standard error of the mean values in parentheses. Data represent at least two independent experiments. To compare mutants to WT, significance was calculated by using one-way ANOVA and Dunnett’s post hoc: *, *p* < 0.05; **, *p* < 0.01, ***p < 0.001; ****, *p* < 0.0001. To compare 24 h to 48 h, significance was calculated using unpaired Student’s *t-*test: ^#^, *p* < 0.05.

Strain	Biomass (μm ^3^ /μm ^2^ )		Average thickness (μm)		Roughness coefficient (R*)		Average particle size (μm)	
Time (hours)	24	48	24	48	24	48	24	48
**WT**	0.66 (0.58)	8.21 (2.57)^#^	24.42 (15.79)	24.07 (12.85)	1.91 (0.05)	0.80 (0.30)^#^	45.74 (11.22)	87.79 (15.60) ^#^
**Δ*bpsA-D***	0.007 (0.004)*	0.009 (0.004)***	8.53 (6.98)	8.43 (2.48)	2.00 (0.0003)	1.99 (.002)*^,#^	2.68 (1.10)****	14.72 (6.00)****^, #^
**Δ*fhaB***	0.006 (0.001)*	0.01 (0.001)***^,#^	6.15 (3.36)	3.56 (1.73)	2.00 (0.0004)	2.00 (0.001)*	3.83 (1.81)****	2.59 (1.19)****
**Δ*cyaA***	ND	3.09 (2.68)*	ND	16.49 (5.20)	ND	1.18 (0.81)*	ND	38.36 (20.03)****

Staining of WT biofilms for Bps and eDNA resulted in the visualization of tripartite complexes consisting of densely packed bacterial cells, Bps, and eDNA at 24 and 48 h post-infection. In addition, bipartite complexes containing Bps and eDNA, as well as individual staining for Bps, eDNA, and bacterial clusters, were observed (Fig 4A–4D). Quantitation of median fluorescence intensity (MFI) of the ECM components of WT biofilms revealed a significant increase in Bps-specific fluorescence from 24 h (731.33±123.24 MFI) to 48 h (1444±49.73 MFI) post-infection (*p <* 0.0001). A slight but not significant increase in eDNA-specific fluorescence was also observed from 24 h (246.67±144.24 MFI) to 48 h (484±387.52 MFI) post-infection (*p* = 0.726) (S3 Fig). We conclude from these data that the two *Bp* ECM components (Bps and eDNA) are (i) associated with the bacterial cells, resulting in a 3D scaffold that includes the bacterial cells and (ii) present extracellularly and form complexes with each other independent of bacterial cells. Altogether, these data show that (i) *Bp* forms structured biofilms on HBE cells and (ii) *Bp* biofilms have an ECM composed of Bps and eDNA.

### Production of Bps and FHA is essential for biofilm formation on HBE cells

**Fig 5:**
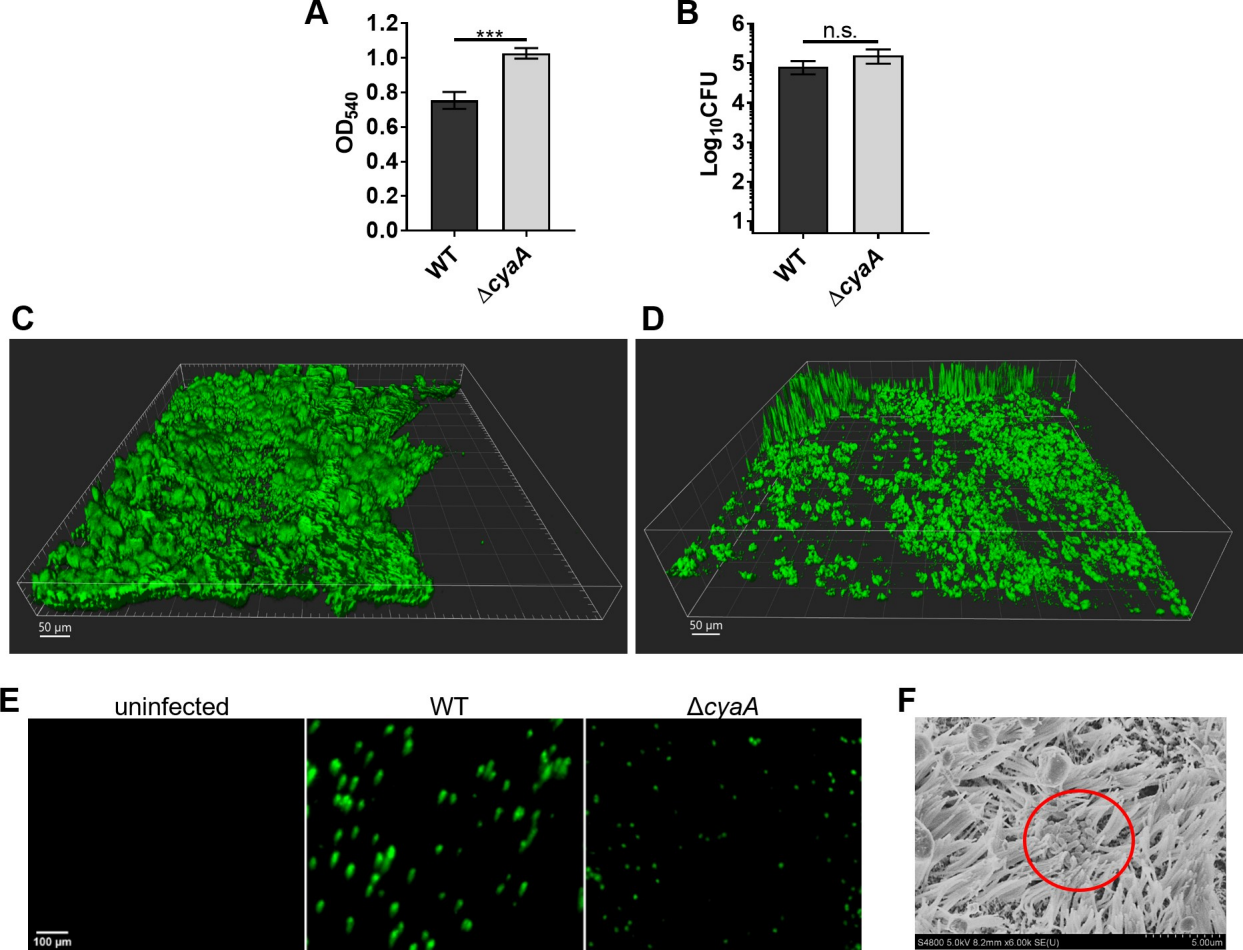
The role of adenylate cyclase toxin in *Bp* biofilm formation on HBE cells. (A) Biofilm formation after 96 h was evaluated by GFP-labeled bacteria in microtiter plates by crystal violet method. Bars indicate the mean and standard error of the mean of two independent experiments performed by triplicate. Significant differences were calculated by using one-way ANOVA and Bonferroni post hoc. ***, *p* < 0.0005. (B-E) HBE cells were apically infected with bacterial strains in DMEM for 1 h at MOI 10. (B) CFUs were enumerated after 24 h on BG agar. Mean and standard error of the mean of two biological replicates in technical duplicate are presented. Statistical differences were assessed by unpaired Student’s *t*-test. n.s., not significant. (C-D) HBE cells infected with GFP-expressing WT (C) or Δ*cyaA* (D) strains for 48 h were fixed and imaged using an Olympus FV3000 confocal microscope (20X objective). Z-stacks were acquired at 1-μm intervals. IMARIS software was used to produce a shading picture of the biofilms. (E) Representative live cell images of GFP-labeled bacteria on apical surface of HBE cells after 48 h (10X objective). (F) Scanning electron microscopy of bacterial strains 24 h after infection of HBE cells. For C-F, images are representative of at least two biological replicates.

In comparison to HBE cells infected with the WT strain, confocal microscopy of HBE cells infected with either the Δ*bpsA-D* or Δ*fhaB* mutants resulted in fewer and smaller bacterial clusters at both 24 and 48 h post-infection (Fig 4E–4L). As expected, infection of HBE cells with the Δ*bpsA-D* strain did not result in staining for Bps, thereby demonstrating the specificity of the antibody (Fig 4E–4H). While some staining for eDNA was observed after 24 h infection with the Δ*bpsA-D* strain, eDNA did not appear to colocalize with bacterial cells. While the majority of the Δ*fhaB* cell clusters formed after 24 h did not colocalize with either Bps or eDNA (Fig 4I–4L), a few bipartite complexes of Bps-eDNA, and some staining for Bps alone, were observed after infection with Δ*fhaB* strain (Fig 5). As expected, the MFI of Bps and eDNA for the mutant strain biofilms was lower than the WT strains (S3 Fig). We conclude from these data that presence of FHA and Bps on the cell surface is critical for biofilm formation on HBE cells.

### Adenylate cyclase toxin (ACT) does not inhibit biofilm formation on HBE cells

Recently, it was shown that a mutant strain of *Bp* deficient in the production of ACT formed higher levels of biofilms compared with the WT strain on plastic surfaces [16]. We confirmed this phenotype by comparing biofilm formation on polystyrene microtiter plates between the WT and Δ*cyaA* (containing an in-frame deletion in *cyaA* gene, encoding ACT [33]) strains (Fig 5A). To examine if ACT plays a role in biofilm formation on human airway epithelium, HBE cells were infected with GFP-expressing derivatives of WT and Δ*cyaA* strains, and biofilm formation 48 h post-infection was evaluated by CLSM. CFUs recovered were comparable between WT and Δ*cyaA* bacteria (Fig 5B), suggesting that ACT does not play a significant role in adherence to HBE cells. Compared to the WT strain, which formed a mat of adhered bacterial cells covering large areas of the HBE apical surface (Fig 5C), the Δ*cyaA* strain formed smaller aggregates with thin spikes of cell clusters, which were dispersed along the HBE apical surface (Fig 5D). The biofilm biomass and the average particle size of the Δ*cyaA* biofilms were approximately 2.6- and 2.2-fold lower, respectively, than that of WT biofilms (Table 2). We obtained comparable results with live immunofluorescent imaging, which showed Δ*cyaA* bacteria forming smaller aggregates than WT bacteria (Fig 5E). Moreover, as shown by SEM, while the Δ*cyaA* cells attached to cilia, as was observed for the WT strain, the aggregates of the Δ*cyaA* strain were smaller than those of WT (compare Figs 5F with 3C). In summary, contrary to its inhibitory role in biofilm formation on abiotic surfaces [16], the absence of ACT production in *Bp* does not enhance biofilm formation on HBE cells but results in biofilms with lower biomass and average particle size, and a higher roughness coefficient.

## Discussion

In 1912, Mallory and Hornor reported ‘masses of minute bacilli’ in the trachea and bronchi of patients who died of whooping cough [34]. In the published drawings, bacterial cells were present in the form of large densely packed aggregates and clusters, a mode of existence for bacteria later termed as ’biofilms.’ About a hundred years later in 2008, bacterial clusters and tangles on the cilia of epithelial cells lining the trachea and bronchioles of patients with fatal pertussis were observed *[35]*. Although the description by Mallory and Hornor represents one of the earliest examples of bacterial biofilms on human organs, experimental research with *Bp* biofilms has lagged.

In this manuscript, we used a model of well-differentiated primary human bronchial epithelial cultures from human donors [36–38]. These HBE cultures were composed of multiple cell types which included ciliated cells, mucus-secreting cells, and intermediate and basal cell types. Additionally, these were polarized, pseudostratified, and presented with healthy tight junctions and functionally beating cilia. The formation of beating cilia is important since a major characteristic of *Bp* infection of humans is specific adherence to ciliated respiratory epithelium [30,39,40], a phenotype observed by us herein upon infection with the WT *Bp* strain. Following infection with isogenic mutant strains containing unmarked in-frame deletions, we found that the presence of FHA and Bps promoted the early attachment of *Bp* to human cilia. While a phenotypic role for FHA in adherence to human airway epithelial cells was previously described [25], a role for Bps in attachment to ciliated human lung epithelium was not known. Previously, we found that Bps was not involved in *Bp* adherence to A549, an immortalized human cell line consisting of alveolar basal epithelial cells [18]. However, Bps was required for efficient attachment to RPMI 2650, an immortalized human nasal epithelial cell line. In addition to lacking many physiologically relevant characteristics, like mucus production and beating cilia, immortalized airway cell lines often lack the expression of specific receptors required for optimal infection [41]. It is possible that A549 cell line does not express or expresses low amounts of a yet to be identified Bps-specific receptor. Therefore, our results newly identify Bps as an adhesin that facilitates initial binding to the human bronchial epithelium, which is the natural niche of *Bp* and emphasize the importance of utilizing primary human airway epithelial cells over immortalized monolayer cell lines for pathogenesis research.

At 24 h post-infection and compared to the Δ*fhaB* strain, there was a greater reduction in the numbers of the Δ*bpsA-D* strain associated with the HBE cells. We have recently discovered that Bps promotes resistance to human anti-microbial peptides (AMPs) [42]. Production of many AMPs by human epithelial cells is induced upon bacterial infection under *in vitro* culture conditions and *in vivo* [43]. Therefore, by contributing to both *Bp* adherence and resistance to AMPs, Bps can enhance the infection of *Bp* in the human airways.

Infection of HBE cells with the WT *Bp* strain resulted in a decrease in TEER, indicating a disruption of barrier integrity. This disruption can lead to leakage of basal media through to the apical surface, increasing the rate of cilia beating, which we observed with the WT and Δ*fhaB* strains. Disruption of the barrier integrity of hamster and human airway epithelial cells due to *Bp* infection has been previously shown to be dependent on the production of tracheal cytotoxin, a product of the *Bp* cell wall and ACT [44–47].

Important advances have been made regarding the interactions between ECM components and bacterial cells during biofilm growth on abiotic surfaces [48–51]. However, processes by which biofilms are formed on human surfaces is poorly understood [52]. Having established that our HBE culture model developed above closely resembles the natural *Bp* infectious environment, we utilized SEM and CLSM to detect bacterial biofilms. Bacterial aggregates, clusters, and highly structured biofilms adherent to cilia were detected. The colocalization of bacterial biofilms with cilia on primary airway cells mimics the observations from pathological analyses of patient lung tissues which is described as formation of bacterial clusters and tangles on ciliated cells [34,35]. Oftentimes, mature biofilms formed on abiotic surfaces are covered by an ECM composed of polysaccharides and eDNA [15]. At many locations on the apical surface of HBE cells, *Bp* cells were covered by both Bps and eDNA, resulting in the formation of tripartite complexes. We also observed bipartite complexes containing both Bps and eDNA, as well as individual structures of eDNA, Bps, or bacterial cells. While some studies have documented the presence of ECM components in primary human cultures and human tissues [53–55], tripartite or bipartite complexes have not yet been documented. In one study, strings of DNA were observed independent of bacterial aggregates formed on lung tissues from chronically infected cystic fibrosis patients [56]. Taken together, our results demonstrate complex heterogeneity in the structure and interactions of biofilm cells and ECM matrix components in a system resembling human infection.

By utilizing isogenic mutant strains, we examined the roles of Bps and FHA in biofilm formation. For both these mutant strains, fewer and smaller clusters of bacteria were found to be associated with HBE cells, suggesting the critical roles that these two factors play in attachment and biofilm formation on human airway epithelia. The clusters and aggregates of the mutant strains largely stained negative for either Bps or eDNA. However, limited staining for eDNA (infection with Δ*bpsA-D* mutant) and for eDNA and Bps (infection with Δ*fhaB* mutant) was observed. Our interpretation from this is that ECM components (Bps and eDNA) become associated with only matured and structured biofilms.

We propose that Bps and eDNA collectively play key structural roles and function in bacterial adhesion, further enhancing the stability of biofilms, which resist the clearing actions of beating cilia. The PNAG/PGA family of polysaccharides, of which Bps is a member, contain variable amounts of de-*N*-acetylated glucosamine residues, rendering these polymers positively charged [57–59]. Thus, Bps produced and released during HBE culture infection could complex with eDNA, resulting in the formation of dual complexes, independent of bacteria. Cationic antimicrobial peptides (AMPs), key components of host airway defense, interact with negatively charged eDNA. We propose that Bps and eDNA, and their complexes, can sequester AMPs produced by HBE cells, thereby preventing them from interacting with the bacterial surface, resulting in protection of *Bp* cells from clearance [42]. We previously showed that Bps promotes resistance to serum-dependent killing and inhibits the deposition of complement [24]. Thus, Bps produced during infection, and as a component of the biofilm ECM, can provide a physical and chemical barrier against host immune components.

In addition to Bps and FHA, we determined the role of ACT in *Bp* biofilm formation on HBE cells. In contrast to its inhibitory role in biofilm formation on abiotic surfaces [16], we found that compared to WT biofilms, the biofilms of the Δ*cyaA* strain had reduced biomass and average particle size, thereby suggesting that ACT positively contributes to mature biofilm formation on HBE cells. Although it has been previously shown that interactions between ACT and FHA lead to biofilm inhibition on abiotic surfaces [16], ACT is also required for the colonization of mouse lungs [60]. ACT is both associated on the cell surface and secreted from *Bp* [61]. *In vitro* conditions utilized for previous examination of the function of ACT in biofilm formation may have resulted in higher levels of ACT in the culture media, facilitating its interaction with FHA and thereby resulting in inhibition of biofilm formation. Additional studies are needed to dissect the precise mechanism by which ACT contributes to biofilm formation on HBE cells.

Infants are highly susceptible to pertussis and often exhibit severe symptoms that are not observed in older children and adults. While the importance of bacterial biofilms in chronic/persistent bacterial infections is well-established, acute infections are predominantly considered to be due to planktonic bacteria. Recently, bacterial biofilms were detected during acute lung infections, necrotizing fasciitis, and acute otitis media [62–65]. While we previously detected *Bp* biofilms in the upper respiratory tract of mice [18,19], *Bp* biofilms could not be detected in murine lungs. Results from this study along with pathological evidence of clusters and tangles in the bronchi of infants who died from pertussis [34,35] suggest that *Bp* utilizes biofilms as a survival strategy within the human lungs.

*Bp* infection of older children and adults and vaccinated individuals results in milder symptoms and often leads to the establishment of an asymptomatic carriage state in the nasopharynx. An increase in the number of cases of asymptomatic infections, transmission, and experimental demonstration of asymptomatic human colonization of *Bp* [9] necessitates detailed understanding of the mechanisms by which *Bp* colonizes the human nasopharynx. Nasopharyngeal biofilms could contribute to the establishment of an asymptomatic carriage state of *Bp* by enhancing its survival against host immunity and providing an *in vivo* nidus for transmission to other hosts and circulate in the community. The HBE cell model developed here should serve as a foundation for studying the contribution of *Bp* biofilms under conditions that mimic the human nasal airway mucosa [66].

## Materials and methods

### Ethics statement

The Nationwide Children’s Institutional Review Board has approved our collection, processing and use of de-identified human tissues from organ donors. Under the 2018 Common Rule, no continuing review is required. The approval ID is STUDY00000314.

### Bacterial strains and growth conditions

*Bp* strains and plasmids used in this study are listed in Table 3. Strains were maintained on Bordet-Gengou (BG) agar [67] supplemented with 10% defibrinated sheep blood (HemoStat, Laboratories) at 37°C for 96 h. For liquid growth, strains were incubated at 37°C for 24 h in a roller drum (80 rpm) in Stainer-Scholte (SS) [27] medium supplemented with 0.1 mg/ml of heptakis (2,6-di-*O*-methyl-β-cyclodextrin, MP biomedicals). When growing bacteria harboring the plasmid pGBSp1 for GFP expression [68], SS medium and BG agar was supplemented with 20 μg/ml kanamycin (Kan). *Burkholderia cenocepacia* (K56-2 strain labeled with DsRed) was grown in Luria-Bertani (LB) broth at 37°C for 24h at 200rpm.

**Table 3:** Strains and Plasmids.

Strain	Characteristics	Reference or Source
WT	Bp536, reference strain; Sm^R^	Laboratory stock
Δ*bpsA-D*	Bp536 derivative containing an in-frame deletion of the *bpsA-D* locus; Sm^R^	[18]
Δ*fhaB*	Bp536 derivative containing an in-frame deletion of the *fhaB* gene; Sm^R^	[19]
Δ*cyaA*	Bp536 derivative containing an in-frame deletion of the *cyaA* gene; Sm^R^	[33]
Plasmid		
pGBSp1	plasmid containing GFP protein; Kan^R^	[68]

### Construction of green fluorescent protein (GFP)-labeled bacteria

Electrocompetent cells of each strain were transformed with 1 μg of pGBSp1 by electroporation [69] and plated for Kan selection on BG agar.

### Human bronchial epithelial (HBE) cultures

Primary human bronchial epithelial cells (HBE) were provided by Nationwide Children’s Hospital Cure Cystic Fibrosis Columbus Epithelial Cell Core (Columbus, OH) without identifiers (exempt status from the Institutional Review Board). Primary HBE cells were cultured as previously reported in our lab [38,70]. Briefly, primary human airway cells were seeded onto 6.5mm Transwell filters (Corning) coated with collagen Type IV (Sigma) with ~50,000 cells. Cells were seeded and fed with ROCK inhibitor-supplemented air-liquid interface (ALI) media on both sides of the membrane until optimal epithelial integrity was reached, as measured by an epithelial voltohmeter (~7 days; ~300 Ohms·cm^2^). Basal medium was replaced with PneumaCult-ALI (Stem Cell Technologies) differentiation medium, and cells were fed three times per week for ~3 weeks until fully differentiated. Cell differentiation and integrity of the bronchial epithelium were confirmed before each experiment. Cultures were also checked for absence of mycoplasma contamination.

### Hematoxylin & Eosin (H&E) and Periodic Acid-Schiff & Alcian Blue (PAS/AB) staining

Primary HBE cultures were either fixed in 4% (v/v) paraformaldehyde (for H&E) or Carnoy’s fixative (for PAS/AB staining) for 24 h, embedded and sectioned (4 μm thickness) for H&E or PAS/AB staining. Images were captured using a Revolve R4 microscope (Echo). Mucin secretion was semi-quantified using a single-blinded assessment of mucin production based on a reference scale of 0 (no mucin) to 3 (excessive mucin). A total of five independently trained non-pathologists scored the PAS/AB images. For each bacterial strain, at least two biological experiments in technical duplicate were performed.

### Scanning electron microscopy (SEM)

At 24 h post-infection, HBE cells infected with *Bp* strains were washed twice with PBS and fixed with 2.5% glutaraldehyde for 72 h at 4°C. Transwell filters were washed with ethanol and processed by critical point drying and sputter coating. Cells were viewed with a *Hitachi* S-4800 high resolution *scanning electron microscope*. For each bacterial strain, two biological experiments in technical duplicate were performed.

### Immunofluorescence staining

At 48 h post-infection, HBE cells were fixed in 4% PFA for 72 h, washed with ice cold PBS, and blocked with 1% bovine serum albumin (BSA) + 10% normal goat serum in PBS + 0.05% Tween-20 (PBS-T). Antibodies used included acetylated-α-tubulin (EMD Millipore #MABT868, 1:100), MUC5AC (clone 45M1 Sigma Aldrich #M5293, 1:100) and zonula occludens-1 (ZO-1 Invitrogen #339194 conjugated to AlexaFluor-594, 1:100) diluted in blocking buffer were added for 3 h at 37°C (whole filter) or 24h at 4°C (cross-sections). Following three washes with PBS and incubation with blocking buffer for 10 min, cells were incubated with secondary antibody α-mouse IgG AF594 (Life Technologies #A11020, 1:500) for 1.5 h at 37°C. Cells were then washed with PBS, and Transwell filters were mounted on glass slides with Prolong Gold antifade with DAPI (Invitrogen). Cells were imaged with an Echo Revolve R4 immunofluorescent microscope (Echo). Quantification of fluorescence was performed using ImageJ software (v1.52a). For each bacterial strain, two biological experiments in technical duplicate were performed.

### Trans-epithelial electrical resistance (TEER)

At 48 h post-infection, 100 μl of pre-warmed (37°C) Hanks balanced salt solution (HBSS, Mg^2+^, Ca^2+^; Stem Cell Technologies) was added to the apical surface of the HBE cultures, and TEER was measured using an EVOM2 epithelial voltohmeter (World Precision Instrument). TEER was expressed as Ohms·cm^2^. For each bacterial strain, at least two biological experiments in technical duplicate were performed. Positive control for TEER included infecting HBE cultures with *B*. *cenocepacia* (K56-2) with 5x10^4^ CFU in 100μL (MOI = 1) of bacterial suspension and incubated for 2 h at 37°C with 5% CO_2_.

### *Bp* infection

Upon HBE cell differentiation, apical surfaces were carefully rinsed with Dulbecco’s Modified Eagle Medium (DMEM, Corning). HBE cells were infected with 5x10^5^ CFU in 100 μl (MOI 10) of bacterial suspension in DMEM and incubated for 1 h at 37°C with 5% CO_2_. Afterward, DMEM and unattached bacteria were removed, and HBE cultures were incubated at air-liquid interface at 37°C for indicated time points.

### Quantifying bacterial attachment to HBE cultures

To enumerate HBE-associated bacteria, HBE cultures were rinsed with HBSS (Stem Cell Technologies) followed by addition of 0.05% Triton X-100 to disrupt HBE cells. Appropriate serial dilutions were plated on BG agar. For each bacterial strain, at least two biological experiments in duplicate were performed.

### Mock infection of bacterial strains in various media sources

A suspension of 5x10^5^ CFU of *Bp* in SS, DMEM, or apical washes obtained from the HBE cells was added to wells of 24-well plates without 6.5mm Transwell filters and incubated for 24 h at 37°C with 5% CO_2_. Appropriate serial dilutions were plated on BG agar. For each bacterial strain, three biological experiments in technical duplicate were performed.

### Ciliary beat frequency (CBF)

A camera with slow-motion capture of 720 pixels at 240 fps (frames-per-second) mounted on the Revolve R4 microscope (Echo) was used to capture CBF. An average of 4 counts per 5 second video was calculated using the Avidemux software (v2.6). The Echo Revolve R4 microscope was mounted on top of a Vibration Isolation platform (Vibe 30-70lb, Newport Corporation), eliminating vibrational interference. For each bacterial strain, at least two biological experiments in technical duplicate were performed.

### Lactate dehydrogenase quantification

Lactate dehydrogenase (LDH) was quantified from basal media using CytoTox-ONE Homogenous Membrane Integrity Assay (Promega). For each bacterial strain, two biological experiments in technical duplicate were performed.

### Live cell imaging of GFP-expressing *Bp*

At 24 and 48 h post-infection, live cell imaging of fluorescent bacteria was captured using the Revolve R4 immunofluorescent microscope (Echo). Three individual images per Transwell filters were captured (10X objective). Quantification of bacteria was done by using ImageJ (v1.52a). For each bacterial strain, at least two biological experiments in technical duplicate were performed.

### Confocal laser scanning microscopy (CLSM)

HBE cells infected with *Bp* were washed twice with PBS and fixed with 4% PFA overnight at 4°C. Three washes were performed with PBS, followed by the addition of 1% BSA in PBS at room temperature for 30 min. After six washes, 100 μl mouse α-dsDNA (Abcam) (2 μg/ml in BSA in 1% BSA) and goat α-dPNAG (gift from Dr. Gerald Pier) (1:500 in 1% BSA) were added to each Transwell filter and incubated overnight at 4°C. The next day, cells were washed six times with PBS and, subsequently, 100 μl donkey α-goat AF555 and goat α-mouse AF647 (2 μg/ml in BSA in 1% BSA) were added to each Transwell filter and incubated for 2 h at room temperature. After six washes, Transwell filters were mounted on slides using ProLong Gold (Sigma). CLSM image acquisition was carried out by using an Olympus FV300 confocal microscope at 0.5 *μm z-*intervals; *xy* and *xz* (20X objective). Three-dimensional reconstructions and shading projections of image stacks were created by using IMARIS software (Bitplane). ImageJ was used to create 8-bits files (.ome) and to calculate mean particle size. Z-stacks images were processed by COMSTAT 2.0 to determine the biomass, average thickness, and roughness coefficient. Median fluorescence intensity (MFI) was calculated for Bps and eDNA by using IMARIS software. For each bacterial strain, at least two biological experiments in duplicate were performed.

### Crystal violet staining

Biofilm formation was evaluated as previously described [14,18,71]. Briefly, 100 μl (~10^8^ CFU) were added to each well of a microtiter plate and incubated shaking at 37°C for 96 h. Every 24 h, spent media was replaced by fresh SS medium. Crystal violet was used to quantify the adhered biomass. For each bacterial strain, two independent experiments were performed in triplicates.

### Statistical analysis

The unpaired Student’s *t*-test, one-way ANOVA, and two-way ANOVA were used to analyze respective data in GraphPad PRISM software v8.0. The mean and standard error of the mean are shown in all figures, and *p*-values were calculated as described in figure legends. *p* < 0.05 was considered statistically significant. Unless stated otherwise, *, *p* < 0.05; **, *p* < 0.005; ***, *p* < 0.0005; not significant (n.s.), *p* > 0.05.

## Supporting information

S1 Fig HBE cultures mimic the human airway. Fully differentiated HBE cells were processed for (A) Hematoxylin & Eosin staining, (B) scanning electron microscopy, or (C-E) immunofluorescent microscopy to visualize top-down staining of (C) acetylated-α-tubulin (cilia) (20X objective), (D) MUC5AC (goblet cells) (20X objective), or cross-sections to visualize (E) acetylated-α-tubulin (cilia) and zonula occludens-1 (ZO-1; tight junctions), and (F) goblet cells (MUC5AC). In C-E, DAPI (blue) was used to visualize nuclei. All images are representative of at least two biological replicates.
(TIF)

S2 Fig Formation of aggregates and biofilm-like clusters on cilia. Immunofluorescent staining of acetylated-α-tubulin (cilia) and DAPI (nuclei) on fixed HBE cells infected with GFP-labeled bacteria after 48 h (20X objective). Images are representative of two biological replicates.
(TIF)

S3 Fig Quantitation of Bps and eDNA from Bp biofilms. Median fluorescent intensity was calculated for each biofilm time point by using IMARIS software. Average ± SE values from at least three representative experiments are shown. Significance was calculated with one-way ANOVA and Tukey pot-hoc, **, *p* < 0.01, n.s., not significant.
(TIF)
