# Supplementary figures and images for: Architecture and matrix assembly determinants of Bordetella pertussis biofilms on primary human airway epithelium
Source: PLoS Pathog. 2023 Feb 23;19(2):e1011193. doi: 10.1371/journal.ppat.1011193 (PMC9990917; doi:10.1371/journal.ppat.1011193)

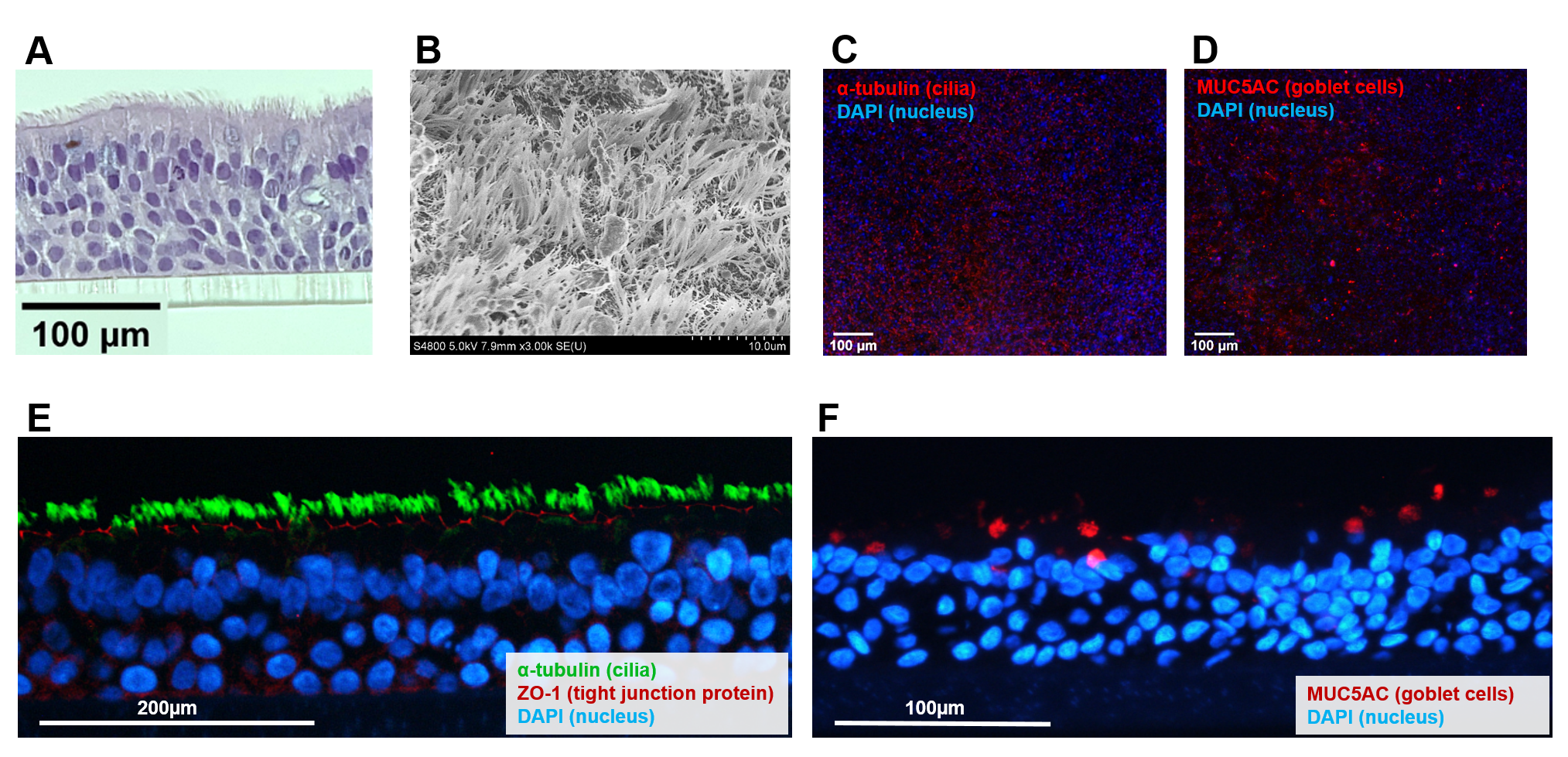

Supplement: S1 Fig — Fully differentiated HBE cells were processed for (A) Hematoxylin & Eosin staining, (B) scanning electron microscopy, or (C-E) immunofluorescent microscopy to visualize top-down staining of (C) acetylated-α-tubulin (cilia) (20X objective), (D) MUC5AC (goblet cells) (20X objective), or cross-sections to visualize (E) acetylated-α-tubulin (cilia) and zonula occludens-1 (ZO-1; tight junctions), and (F) goblet cells (MUC5AC). In C-E, DAPI (blue) was used to visualize nuclei. All images are representative of at least two biological replicates. (TIF) [file ppat.1011193.s001.tif]

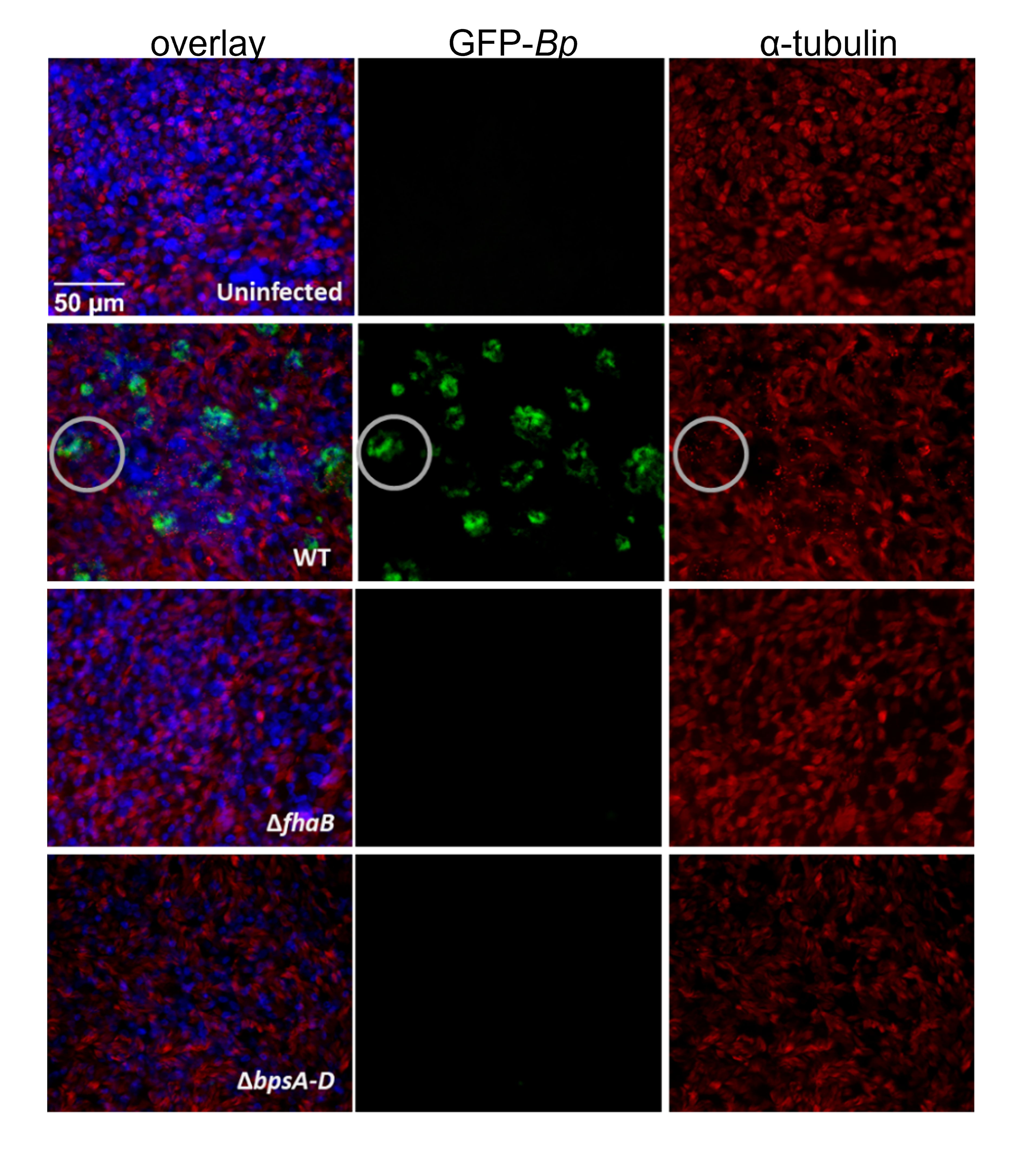

Supplement: S2 Fig — Immunofluorescent staining of acetylated-α-tubulin (cilia) and DAPI (nuclei) on fixed HBE cells infected with GFP-labeled bacteria after 48 h (20X objective). Images are representative of two biological replicates. (TIF) [file ppat.1011193.s002.tif]

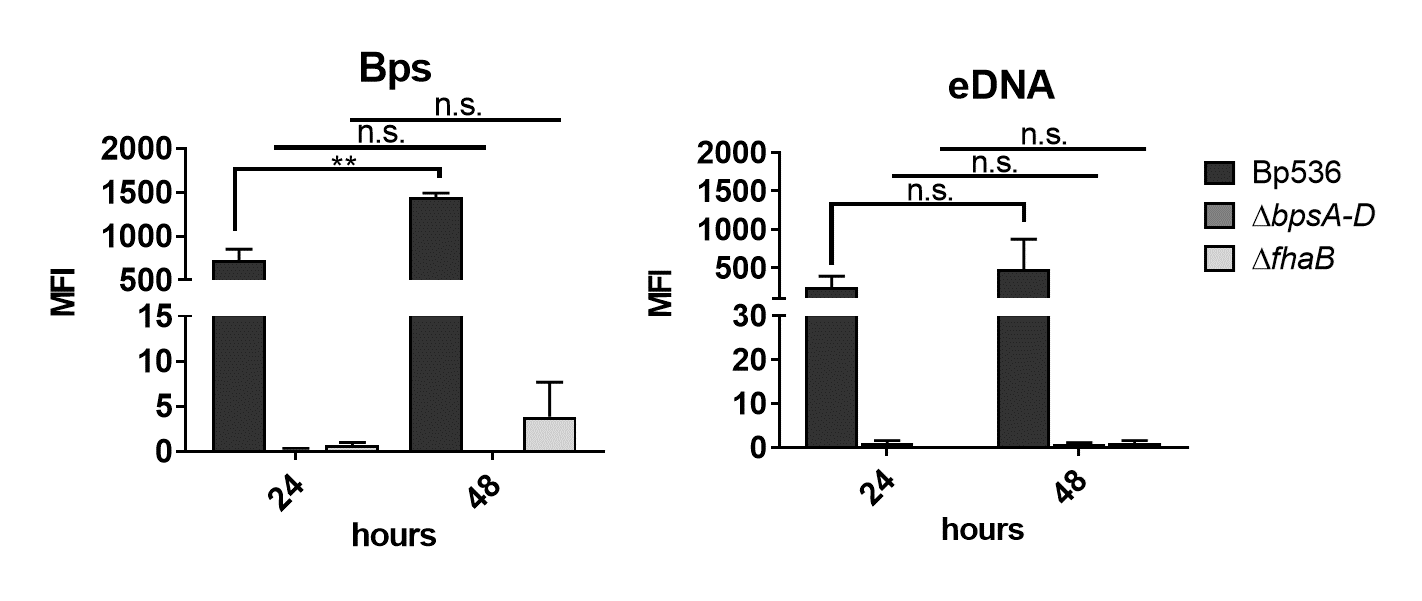

Supplement: S3 Fig — Median fluorescent intensity was calculated for each biofilm time point by using IMARIS software. Average ± SE values from at least three representative experiments are shown. Significance was calculated with one-way ANOVA and Tukey pot-hoc, **, p < 0.01, n.s., not significant. (TIF) [file ppat.1011193.s003.tif]
